# Suppression of Human Tenon Fibroblast Cell Proliferation by Lentivirus-Mediated VEGF Small Hairpin RNA

**DOI:** 10.1155/2017/7982051

**Published:** 2017-01-11

**Authors:** Zhongqiu Li, Wen Hua, Xuedong Li, Wei Wang

**Affiliations:** Department of Ophthalmology, Chaoyang Hospital, Capital Medical University, Beijing, China

## Abstract

*Purpose*. The functions of vascular endothelial growth factor (VEGF) in scar formation after trabeculectomy were investigated in a human Tenon fibroblast cell line from glaucoma patients using lentivirus-mediated VEGF shRNA.* Methods*. Human Tenon fibroblast (HTF) cells were isolated from scar tissue of glaucoma patients during secondary surgery. Lentivirus-VEGF-shRNA was constructed and transfected into HTF cells. Subsequently, VEGF mRNA and protein expression were analyzed using quantitative RT-PCR and western blotting, respectively, and the effects of VEGF knockdown were analyzed. The inhibition of HTF proliferation was monitored according to total cell numbers using ScanArray.* Results*. Both mRNA and protein levels of VEGF were reduced by lentivirus-mediated VEGF-shRNA, and proliferation of HTF cells was inhibited.* Conclusions*. Primary cultures of human Tenon fibroblast (HTF) were established, and proliferation was decreased following inhibition of VEGF. VEGF may be a suitable therapeutic target for reducing scar tissue formation in glaucoma patients after filtration surgery.

## 1. Introduction

Glaucoma is a common cause of blindness, and filtration surgery remains the most effective therapy for reducing intraocular pressure (IOP). Bleb scarring is the leading cause of surgical failure [1], and corticosteroids and antimetabolites, such as triamcinolone, mitomycin C (MMC), and 5-fluorouracil (5-FU), are currently used to inhibit the proliferation of fibroblasts and to increase success rates of surgery [2]. However, the side effects of intraocular toxicity, bleed leakage, postoperative shallow anterior chamber, and necrosis of conjunctiva and sclera warrant studies of safer, more targeted, and effective antifibrosis agents [3, 4].

Vascular endothelial growth factor (VEGF) plays an important role in scar formation. In a previous study, we showed that VEGF levels were significantly increased in the aqueous humor of the anterior chamber of primary open angular glaucomas (POAG) in patients and rabbits at surgery. Expression of the VEGF receptors VEGFR-1 (Flt-a) and VEGFR-2 (KDR/Flk-1) in Tenon fibroblasts was increased and fibroblast proliferation was stimulated following* in vitro* delivery of VEGF. This suggested that Tenon fibroblasts are direct targets of VEGF during scar formation after filtration surgery [5]. Moreover, higher VEGF expression in Tenon tissue was associated with failure of surgery [6], indicating that isoforms of VEGF play differing roles in scar formation after filtration surgery [7].

The use of anti-VEGF agents to control scar formation after trabeculectomy remains controversial [8–12]. How et al. used the VEGF monoclonal antibody bevacizumab with 5-FU injections into the subconjunctiva to inhibit experimental surgical glaucoma scarring [13]. Nilforushan et al. found that both trabeculectomy with subconjunctival bevacizumab and MMC are effective in controlling IOP profiles. However, the effects of adjunctive subconjunctival bevacizumab were less prominent than those of MMC [14]. In a randomized, controlled clinical trial, local conjunctival necrosis was reported in the subconjunctival bevacizumab group [15]. Contraindications, such as pregnancy, breast feeding, and uncontrolled systemic hypertension, are also important considerations when using bevacizumab. Recently, ranibizumab was used as an anti-VEGF agent after filtration surgery and led to severe hypotony and bleb leak [16], indicating the requirement of safer, more potent anti-VEGF agents.

RNA interference (RNAi) has emerged as a powerful tool to induce loss-of-function phenotypes by posttranscriptional silencing of gene expression and has been used to suppress VEGF-induced retinal neovascularization [17]. In the present study, we used a lentiviral vector expressing a small hairpin RNA (shRNA) to inhibit the expression of VEGF in human Tenon fibroblast (HTF) cells.

## 2. Material and Methods

The tenets of the Declaration of Helsinki were upheld, approval was granted by the IRB committee, and informed consent was obtained for all human experiments.

### 2.1. Human Tenon Fibroblast Isolation and Quality Control

Scar tissues were obtained from eight bleb-scarring patients who had secondary surgeries after trabeculectomy. There was no difference in terms of harvesting HTF between the patients with the primary and secondary surgeries. Primary cells were cultured as tissue adherence explants. For series quality control, observations of cell morphology, immunofluorescence cell validation using mouse antihuman vimentin monoclonal antibody (ZF-0512, Zhongbin Golden), mycoplasma testing (Huyuan Biotech), and stability testing were performed and growth curves, proliferation, and population doubling times were determined in isolated primary cells. Primary HTF cells were mycoplasma free and no significant changes in cell morphology or growth rates were observed between passage 5 and passage 15 cells.

### 2.2. Lentivirus Vectors for VEGF Small Hairpin RNA

Five VEGF probes were designed and synthesized based on the released sequence from RNAi Codex as follows:

shRNA-1: 5′-CCGGGCGCAAGAAATCCCGGTATAACTCGAGTTATACCGGGATTTCTTGCGCTTTTT-3′
shRNA-2: 5′-CCGGGACGTGTAAATGTTCCTGCAACTCGAGTTGCAGGAACATTTACACGTCTTTTT-3′
shRNA-3: 5′-CCGGATGCGGATCAAACCTCACCAACTCGAGTTGGTGAGGTTTGATCCGCATTTTTT-3′
shRNA-4: 5′-CCGGCAAGATCCGCAGACGTGTAAACTCGAGTTTACACGTCTGCGGATCTTGTTTTTT-3′
shRNA-5: 5′-CCGGCAAGATCCGCAGACGTGTAAACTCGAGTTTACACGTCTGCGGATCTTGTTTTTT-3′

Positive control miR-214 was designed according to the methods described in Yang et al. [18]. Subsequently, miR-shRNA probes were inserted into pEn-TmiRC3 vectors using PCR and were cloned into pSLIK-Zeo using LR recombination methods. Transformations of the LR reaction were performed using DH10B and recombinant vectors were confirmed using Sanger sequencing.

### 2.3. HTF Cell Transfection and RNAi Efficacy

Recombinant VEGF-shRNA plasmids were transiently transfected into HTF cells, and shRNAs were screened using a pSLIK miRNA-based vector that lacked VEGF interference sequences as a negative control.

Both transiently and stably infected cell lines were grown in 6-well plates with no selection of drug for validation using qRT-PCR and western blotting. DOX was added to cell cultures at a final contraction of 1 *μ*g/mL and the cells were incubated for at least 72 h as necessary. The cells were harvested at indicated times after induction, and cell extracts were prepared using RNeasy Kits (Qiagen) for qRT-PCR or using RIPA buffer for western blotting. Standard protocols were used for qRT-PCR and western blot analyses.

All five VEGF shRNAs reduced VEGF mRNA expression in HTF cells, and shRNA-2 and shRNA-3a reduced expression to 41.0% and 36.5%, respectively, and were selected for subsequent VEGF functional knockdown experiments.

### 2.4. Cell Culture and Transfection

Lenti-neo-GFP with the same backbone as pSLIK-VEGF-shRNA was used as the control titer. Viruses with recombinant target sequences and Lenti-neo-GFP were produced in parallel. Virus titers were determined using FACs analysis, and lentiviral titers of pSLIK-neo-GFP were 1.3 × 10^5^ TU/mL.

Primary HTF cells were cultured in Dulbecco's Modified Eagle Medium containing high glucose (Gibco BRL) and 10% fetal bovine serum. Cells were incubated in a humidified incubator at 37°C in 5% CO_2_ and 95% air. HTF cells were infected with lentivirus-shRNAs at MOI = 1. At 96 h after transfection, 1 *μ*g/mL of DOX was added to the cultures to induce the transcription of shRNA.

### 2.5. Western Blot Analysis

At four days after induction with DOX, transfected HTF cells were collected and lysed in buffer containing 150 mM NaCl, 50 mM Tris-HCl (pH 7.4), 2 mM EDTA, 1% NP-40, and protease inhibitors (Boehringer Mannheim, Germany). Total protein in extracts was measured using the Bradford method. Aliquots of total proteins were resolved using SDS polyacrylamide gel electrophoresis and were then transferred onto immobilon P membrane using an SD semidry transfer apparatus according to the manufacturer's instructions. Membranes were incubated with polyclonal antihuman VEGF antibody (Cat. #AF-293-NA, R&D Systems, USA) and monoclonal antihuman *β*-actin (1 : 10,000 dilution; Sigma). Peroxidase-conjugated secondary antibodies were used as secondary detection reagents with an enhanced chemiluminescence kit (Cat. #20-500, Biological Industries, Israel). X-ray films were developed to visualize chemiluminescence signals.

### 2.6. Proliferation Experiments in HTF Cells after VEGF Knockdown

HTF cells were seeded into 96-black well plates at 30,000 cells per well. Cells were then infected with lentiviru-shRNA-2 and shRNA-3. After four days of induction with DOX, cell quantities were determined using ArrayScan and curves were calculated using PRISMBIOLAB.

## 3. Results

### 3.1. Comparisons of Interference Efficacy between Designs of Lentivirus-shRNA

Interference efficiencies were compared between various transfected VEGF-shRNA plasmid constructs in cultured HTF cells. VEGF mRNA levels were measured using quantitative RT-PCR.

**Figure 1:**
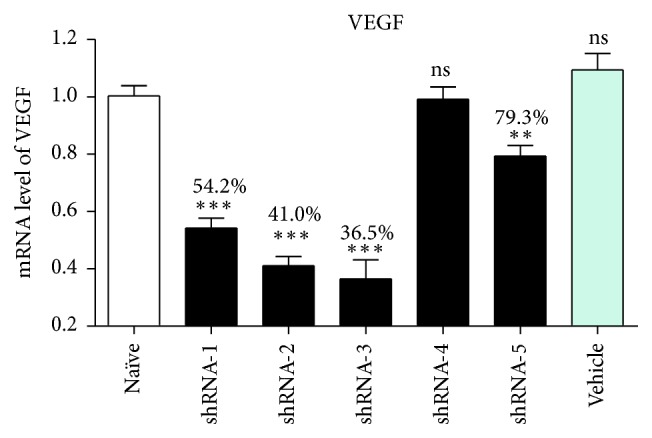
RNA interference after transfection with VEGF-shRNA. ^*∗∗*^ *p* < 0.1; ^*∗∗∗*^ *p* < 0.01.

“Naïve” indicates relative VEGF mRNA expression in nontransfected controls and vehicle indicates that of samples transfected with vehicle only. With the exception of shRNA-4, all VEGF-shRNAs significantly reduced VEGF mRNA levels, and VEGF-shRNA-2 and VEGF-shRNA-3 inhibited expression to 41.0% and 36.5%, respectively, and were selected for functional analyses; see Figure 1.

### 3.2. Suppression of VEGF Expression by VEGF-shRNA-2 and VEGF-shRNA-3

**Figure 2:**
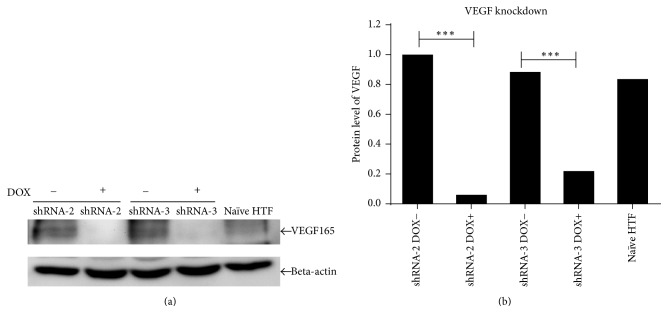
Western blot analysis of the effects of RNA interference on VEGF expression in HTF cells. (a) Relative expression of VEGF in cells treated with shRNA-2 and shRNA-3; (b) protein expression levels in treated groups and naïve HTF controls. ^*∗∗∗*^ *p* < 0.01.

Lentivirus-carrying VEGF-shRNA-2 and VEGF-shRNA-3 were transfected into cultured HTF cells. VEGF protein expression was then investigated using western blotting, which showed reduced VEGF protein expression in HTF cells transfected with lentivirus VEGF-shRNA (Figure 2).

In these experiments, shRNA-2 and shRNA-3 significantly knocked down VEGF protein expression after 96 h of DOX treatment.

### 3.3. Suppression of HTF Proliferation by Lentivirus Transfected VEGF shRNA

Lentivirus-VEGF was transfected into cultured HTF cells. After 96 h, DOX was added to the culture medium to induce the transcription of shRNA.

**Figure 3:**
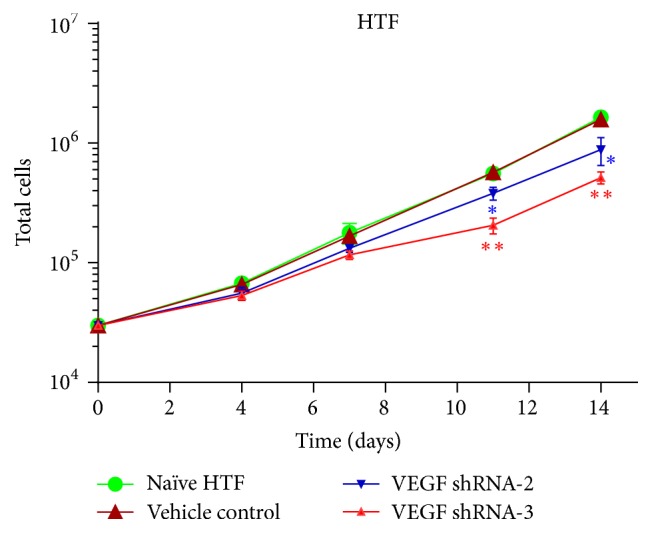
Cell growth curves. Total cell numbers were counted at 4, 7, 11, and 14 days after addition of DOX to cultures. Curves represent total cell numbers of naïve HTF cells, vehicle transfected HTF cells, VEGF-shRNA-2 transfected HTF cells, and VEGF-shRNA-3 transfected HTF cells. ⁎ means *p* value < 0.5 compared to vehicle control. ⁎⁎ means *p* value < 0.1 compared to vehicle control.

Numbers of vehicle transfected HTF cells did not significantly differ from those of naïve HTF cells. However, total numbers of VEGF-shRNA-2 and VEGF-shRNA-3 transfected HTF cells were suppressed, particularly with VEGF-shRNA-3; see Figure 3.

## 4. Discussion

Filtration surgery remains the most effective choice for glaucoma patients. However, excessive scarring remains a leading cause of surgical failure [19–21]. Antimitotic agents, such as mitomycin C (MMC) and 5-fluorouracil (5-FU), have been used to prevent postsurgical scarring and improve surgical outcomes. However, nonselective cell death and apoptosis caused by these agents may lead to serious complications and limit their use [22–24].

Among wound healing factors, angiogenesis plays a key role and VEGF is a key factor because it stimulates angiogenesis, inflammation, and fibrosis at operative sites in glaucoma patients. Several VEGF isoforms and three high affinity VEGF receptors have been identified [25]. In particular, VEGF-R2 mediates most VEGF related-biological responses, and anti-VEGF agents, such as monoclonal antibodies and VEGF traps, have been used as antiangiogenic treatments [26–30]. Previously, we showed that VEGF receptors are expressed in human and rabbit Tenon fibroblasts, which are the main effector cells of ocular scar formation after filtration surgery. At filtration sites, VEGF modified fibroblast activity and caused collagen deposition and contraction, leading to scar formation; moreover, VEGF stimulated Tenon fibroblast proliferation* in vitro*. Accordingly, bevacizumab has been administered to inhibit Tenon fibroblast proliferation but requires repetitive injections [5]. The main complication of trabeculectomy is postoperative scar formation. We found that in our clinic, even with the same operator and same procedures, some patients had postoperative scars while others did not have. Regarding this and comparing with our previous study, we collected the Tenon tissues from patients with secondary surgeries in this study. Although systemic administration of bevacizumab for certain cancers has reported side effects, such as proteinuria, edema, and hypertension, intravitreal usage of bevacizumab has been reported as effective and safe [31–33]. However, long-term safety remains unclear, and RPE tears and other complications have been reported in treated patients [34, 35].

In the present study, we demonstrated that inhibition of VEGF expression using lentivirus-mediated shRNA suppresses the proliferation of human Tenon fibroblast cells. These observations may reflect binding of VEGF to VEGFR-2, leading to activation of the MAPK/ERK pathway, which is a key signaling pathway of cell proliferation, differentiation, and apoptosis. VEGFR-2 dimerization activates the intracellular serine/threonine kinase Raf, the dual specificity MAPK kinase (MEK), and the MAPK-ERK pathway upon stimulation, leading to increased DNA synthesis and cell proliferation. In addition, inhibition of ERK phosphorylation limits proliferation of Tenon fibroblasts, indicating that VEGF stimulates the proliferation of Tenon fibroblasts via the MAPK/ERK pathway. Phosphorylated ERK is translocated into the nucleus and phosphorylates growth factors, such as c-fos, c-Jun, c-myc, and ATF2, which lead to increased nucleotide synthesis, activated transcription and translation, and enhanced cell cycle progression [36, 37]. VEGF has also been shown to affect TGF-beta1/Smad/Snail pathways and trigger myofibroblast transformation [38].

We used lentivirus-mediated VEGF-shRNA to suppress VEGF mRNA expression. Previous studies of RNAi for VEGF show inhibition of neovascularization in ocular angiogenic diseases and demonstrate significant specific inhibition of VEGF by siRNAs [17, 39]. However, to the best of our knowledge, the present data are the first to show that inhibition with VEGF-shRNA also inhibits fibrosis. RNAi is a powerful emerging tool for gene knockdown, and since the 1990s, more than 50 RNA and RNA-derived therapeutics have reached clinical trials [40].

Efficiency and specificity of delivery represent a significant obstacle to therapeutic RNAi. In the present study, a lentivirus-mediated VEGF-shRNA system was used for the following reasons: lentiviruses efficiently transfect cells [41], lentiviral delivery of siRNA is suitable for local administration in ocular tissue, and the vector is transcribed in a DOX-dependent manner allowing easy control of shRNA transcription. Off-target effects of* in vivo* siRNA delivery are another obstacle to siRNA therapies. However, proprietary modifications of siRNA QPI-1007 structure and chemistry have been reported, with similar drug efficacy and reduced off-target effects [42].

The main surgical failure of using MMC or 5-FU is bleb leaking, which is followed by complications as infections and hypotony-related maculopathy. In the previous study, we observed the postoperative effect of inhibiting VEGF in rabbit model. In addition, no bleb leakages were found. In the future study, we will research on lentivirus VEGF-shRNA inhibiting scarring after trabeculectomy in animal models. The comparative research will be also designed in antiproliferative effects between shRNA and MMC.

In summary, lentivirus VEGF-shRNA was successfully used to knock down VEGF expression in Tenon fibroblasts from patient glaucomas, and fibroblast proliferation was suppressed. Subsequent inhibition of ERK phosphorylation had a similar effect, indicating that VEGF stimulates Tenon fibroblast proliferation via the MAPK/ERK pathway. RNAi technology may produce an adjunct agent that improves outcomes of glaucoma filtration surgery, especially secondary surgery for patient with postoperative scar formation.
